# Association between ultraviolet radiation exposure dose and cataract in Han people living in China and Taiwan: A cross-sectional study

**DOI:** 10.1371/journal.pone.0215338

**Published:** 2019-04-25

**Authors:** Hisanori Miyashita, Natsuko Hatsusaka, Eri Shibuya, Norihiro Mita, Mai Yamazaki, Teppei Shibata, Hidetoshi Ishida, Yuki Ukai, Eri Kubo, Hiroshi Sasaki

**Affiliations:** Department of Ophthalmology, Kanazawa Medical University, Uchinada, Japan; Central Research Institute of Electric Power Industry (CRIEPI), JAPAN

## Abstract

**Purpose:**

We investigated associations between ocular ultraviolet (UV) radiation exposure dose and cataract opacities among Han people living in China and Taiwan, to assess the effects of UV exposure intensity.

**Methods:**

This cross-sectional study included Han people aged ≥40 years (1,801 individuals, 450 in Sanya, 636 in Taiyuan, and 715 in Taichung) as subjects who completed a questionnaire including items about diabetes, smoking, steroid use, work history, and time spent outdoors, and underwent an ophthalmic examination. Right eye axial length was measured using A-mode ultrasonography or IOLMaster. Slit-lamp imaging under maximum mydriasis was used to classify cataracts into three major types [cortical (COR), nuclear (NUC), and posterior subcapsular cataracts (PSC)] and two subtypes [retrodots (RD) and waterclefts (WC)] by one ophthalmologist. COR was divided into opacity presence (CEN+) or absence (CEN-) in the central 3-mm diameter area of the pupil. COR was also subdivided into three groups according to opacity shape: axle-shaped opacity concomitant with WC, wedge-shaped opacity around the pupil to the eye center, and ring-shaped opacity in the lens equator along the pupillary margin. The cumulative ocular UV exposure (COUV) was calculated. A logistic regression analysis was used for multivariate analysis.

**Results:**

Cataract odds ratios in high COUV eyes were 5.35 for NUC, 1.87 for PSC, and 1.35 for RD. In eyes with WC, risk of COR ring-shaped opacity significantly increased but that of wedge-shaped opacity (CEN+) significantly decreased. In eyes without WC, risk of COR axle-shaped opacity (CEN–) and ring-shaped opacity significantly increased but that of wedge-shaped opacity (CEN+) significantly decreased.

**Conclusions:**

Increased COUV level among Han people may be a risk factor for the development of nuclear cataracts, PSC, retrodots and ring-shaped cortical cataract. Risk of ocular UV exposure for cortical cataract may differ by opacity shape.

## Introduction

Many studies have reported ultraviolet (UV) radiation to be a risk factor for cataract [1–3]. Although the onset of anterior cortical cataract has been reported to be caused by UVB exposure through some animal experiments [4–7], most reports have assessed the application of much stronger UVB exposure than the normal daily exposure dose. To the best of our knowledge, no reports have used an animal model to investigate the effects of long-term exposure to low-dose UVB. Giblin et al. previously reported on how long-term exposure to low-dose UVA induced the development of nuclear cataract (NUC) in a guinea pig model [8]. Although many previous epidemiological studies have reported an association between cortical cataract (COR) and UV light [9–11], most of these studies were conducted in regions ranging from mid- to high-latitudes. To date, only a few studies have been conducted in low latitude regions with strong UV exposure. Some studies investigating NUC have reported no association with UV exposure [9, 12], in contrast, others have reported relationships between these factors [13–17]. In addition, some reports have indicated an association between UV exposure and posterior subcapsular cataract (PSC) [18]. Retrodots (RD) and waterclefts (WC) are cataract subtypes that cause decreased visual function, with a high prevalence among middle-aged to elderly individuals [19, 20]. Because no studies have investigated associations between UV exposure and these types of cataract, their relationship with UV exposure remains unknown. Moreover, few reports have examined this relationship in a single race living in regions with different UV intensities.

In this study, we investigated right eyes of Han people living in three regions ranging from mid- to low-latitude that experienced different UV intensities (Hainan Province and Shanxi Province of the People’s Republic of China, and Taichung City of Taiwan) to assess associations between ocular UV exposure dose and five types of cataract.

## Materials and methods

### Subjects

The study population were rural low-income farmers in Sanya, Hainan, and Taiyuan, Shanxi, of China and urban residents in Taichung, Taiwan. We extracted data only of Han people among subjects of previous epidemiological studies [21–23] which targeted all general residents aged 50 years or older in one village of each Sanya and Taiyuan in China, and general residents aged 40 years or older in Taichung in Taiwan. The original studies excluded only people that were unavailable due to migration or sickness, and those with a history of eye diseases. The present study population comprised 1,801 individuals (right eyes), including 450 individuals living in a rural area of Sanya City, Hainan Province, People’s Republic of China (18°15’ N, 109°30’ E, performed March 5–12, 2006), 636 living in a rural area of Taiyuan, Shanxi Province, People’s Republic of China (37°52’ N, 112°33’ E, performed July 14–21, 2006), and 715 living in an urban area of Taichung, Taiwan (24°9’ N, 120°40’ E, performed July 2–12, 2009). All subjects provided written informed consent. The study adhered to the tenets of the Declaration of Helsinki and the protocol was approved by the Kanazawa Medical University Institutional Review Board.

### Cataract judgement and collection of other data

**Fig 1:**
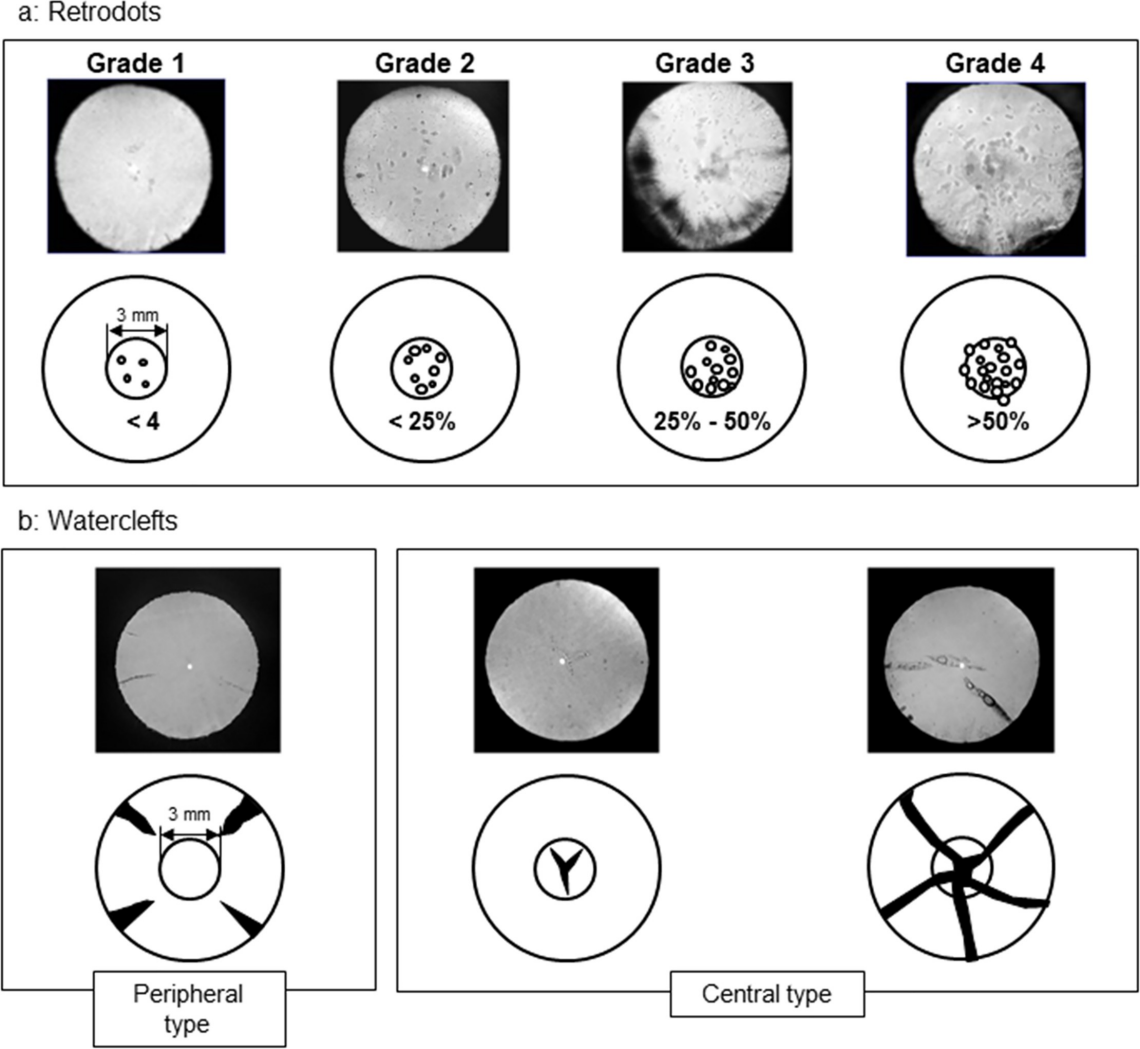
Kanazawa Medical University Cataract Classification and Grading System. a: Retrodots—Judged by size of area of opacity within the central 3-mm diameter area of the pupil, b: Waterclefts—Central type WC: opacity inside but not outside the central 3-mm diameter area of the pupil, Peripheral type WC: opacity outside but not inside this central 3-mm diameter area.

Cataracts were diagnosed by one ophthalmologist from slit-lamp images of right eyes obtained under maximal mydriasis and classified into the three major types (COR, NUC, and PSC) using the WHO classification system [24] and two subtypes (RD and WC) by Kanazawa Medical University Cataract Classification and Grading System (Fig 1).

Other data that are shown in the tables were collected by questionnaire including items about diabetes, smoking, steroid use, work history, and time spent outdoors; and also collected, in interviews conducted by local ophthalmologists, nurses, and ophthalmic or optical residents, who received lectures and exercises on the basics of the epidemiological survey prior to screening.

**Fig 2:**
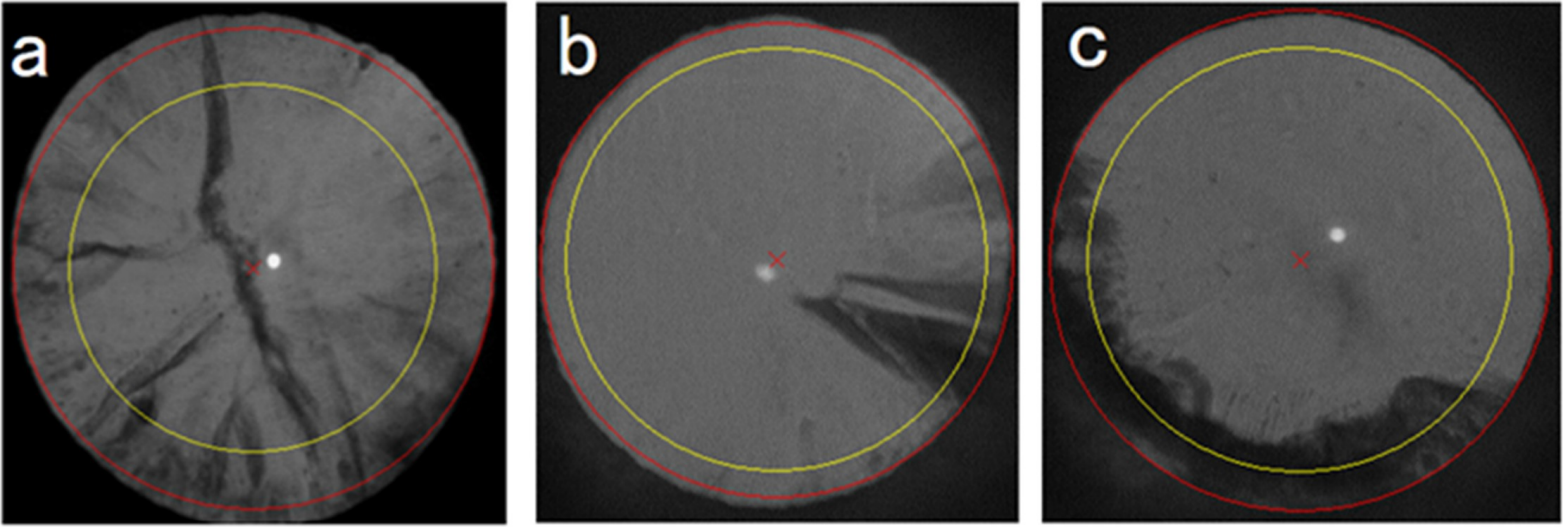
Cortical cataract opacity cases. a: axle-shaped opacity, b: wedge-shaped opacity, c: ring-shaped opacity. Inner circle (yellow) shows the central 6-mm diameter area of the pupil.

COR was divided into opacity presence (CEN+) or absence (CEN-) in the central 3-mm diameter area of the pupil. COR was also subdivided into three groups according to opacity shape: axle-shaped opacity concomitant with WC, wedge-shaped opacity around the pupil to the eye center, and ring-shaped opacity in the lens equator along the pupillary margin (Fig 2). Axial length (AL), a factor of cataract onset, was measured using A-mode ultrasonography (NIDEK Inc., Fremont, CA) or IOLMaster^TM^ (Carl Zeiss AG, Oberkochen, Germany). A previous study reported that the mean AL measured by IOL Master was, on an average, approximately 0.2 mm longer than that measured by A-mode ultrasonography [25]; therefore, for the regions using A-mode ultrasonography, 0.2 mm was added to each case. Other data were collected by questionnaire including items about diabetes, smoking, steroid use, work history, and time spent outdoors.

### Calculation of cumulative ocular UV exposure (COUV)

Previous epidemiological studies used questionnaires to investigate work history, time spent outdoors [21–23], and frequency of the use of hats and sunglasses to determine the degree of ocular UV exposure [10, 26, 27]. Similarly, we collected data of where subjects lived from the age of 20 to the present (ambient UV intensity), time spent outdoors (weekdays and holidays), and whether and how often they used hats, glasses, or sunglasses while outdoors. Based on these data, we calculated their COUV level from the age of 20 to the present using the estimation formula reported by Ono et al. [28].

To calculate ocular UV exposure per day (OUV_day_), “Gl_t_” represented the coefficient for the use of glasses or sunglasses, resulting in a UV cut-off rate of 0.9 for subtraction. Constant use had a coefficient of 1, occasional use had 0.5, and no use had 0. “Hat_t_” represented the coefficient for the use of a hat, resulting in a UV cut-off rate of 0.2 for subtraction. Constant use had a coefficient of 1, occasional use had 0.5, and no use had 0. These values were calculated for the outdoor time reported for weekdays and weekends/holidays. The UV exposure dose for 1 week was calculated based on five weekdays and two weekend days; the mean daily value of the weekly UV exposure was considered the OUV_day_. UV intensity (UV-AB) by region was multiplied by the calculated OUV_day_ values, and COUV was calculated by adding the number of days from the age of 20 to present. According to the National Aeronautics and Space Administration data, UV intensity by region was found to be 233 J/m^2^ in Sanya, 142 J/m^2^ in Taiyuan, and 213 J/m^2^ in Taichung [29].

- Daily ocular UV exposure (OUV_day_)

$${OUV}_{day}={UV}_{day}\times (1-0.9\times {Gl}_{t})\times (1-0.2\times {Hat}_{t})$$

UV_day_: daily UV irradiance
Protection factor of glasses: 0.9
Gl_t_ (glasses use): always = 1, seldom = 0.5, no = 0
Protection factor of hat: 0.2
Hat_t_ (hat use): always = 1, seldom = 0.5, no = 0
  - Mean daily ocular UV exposure (OUV_day_ ave)

$${OUV}_{day}\;ave=[5\times {OUV}_{day}\;(weekdays)+2\times {OUV}_{day}(weekend\;days)]÷7$$

- Cumulative ocular UV exposure (COUV)

$$COUV=\sum_{y=20}^{Age}\left[Ly\sum_{d=0}^{365}\left({OUV}_{day}\;ave\right)\right]$$

COUV: estimated cumulative ocular UV exposure
Ly = Location factor

### Statistical analysis

SPSS Statistics 24 (IBM) was used to perform statistical analysis. A logistic regression analysis was used for multivariate analysis. We investigated the risk of developing the five types of cataract resulting from COUV; age, sex, AL, and history of diabetes were considered as confounding factors.

## Results and discussion

### Subjects distribution

Table 1 presents the number of subjects and the mean ages stratified by sex for each region.

**Table 1:** Number and mean age of the subjects in each region.

	Sanya		Taiyuan		Taichung		Overall	
Sex	M	F	M	F	M	F	M	F
Number of individuals	164	286	240	396	296	419	700	1,101
Age (y)	60.0±9.7	60.1±10.8	62.3±9.5	59.3±8.4	56.0±10.9	54.4±9.6	59.1±10.5	57.7±9.8

### Prevalence of the five types of cataract

Table 2 summarizes the prevalence of the five types of cataract stratified by region.

**Table 2:** Prevalence of the five types of cataract per region.

	Taiyuan UV intensity: 142 J/m^2^	Taiyuan UV intensity: 142 J/m^2^	Taichung UV intensity: 213 J/m^2^
COR (CEN−)	17.6% (14.0–21.1)	12.9% (10.3–15.5)	8.3% (6.2–10.3)
COR (CEN+)	7.6% (5.1–21.1)	10.1% (7.7–15.5)	6.7% (4.9–8.5)
NUC	43.6% (39.0–21.1)	7.2% (5.2–15.5)	6.6% (4.8–8.4)
PSC	12.9% (9.8–21.1)	3.0% (1.6–15.5)	3.4% (2.0–4.7)
RD	40.7% (36.1–21.1)	22.3% (19.1–15.5)	16.2% (13.5–18.9)
WC	4.7% (2.7–21.1)	7.2% (5.2–15.5)	11.9% (9.5–14.3)

() = 95% confidence interval for overall prevalence

### COUV

**Fig 3:**
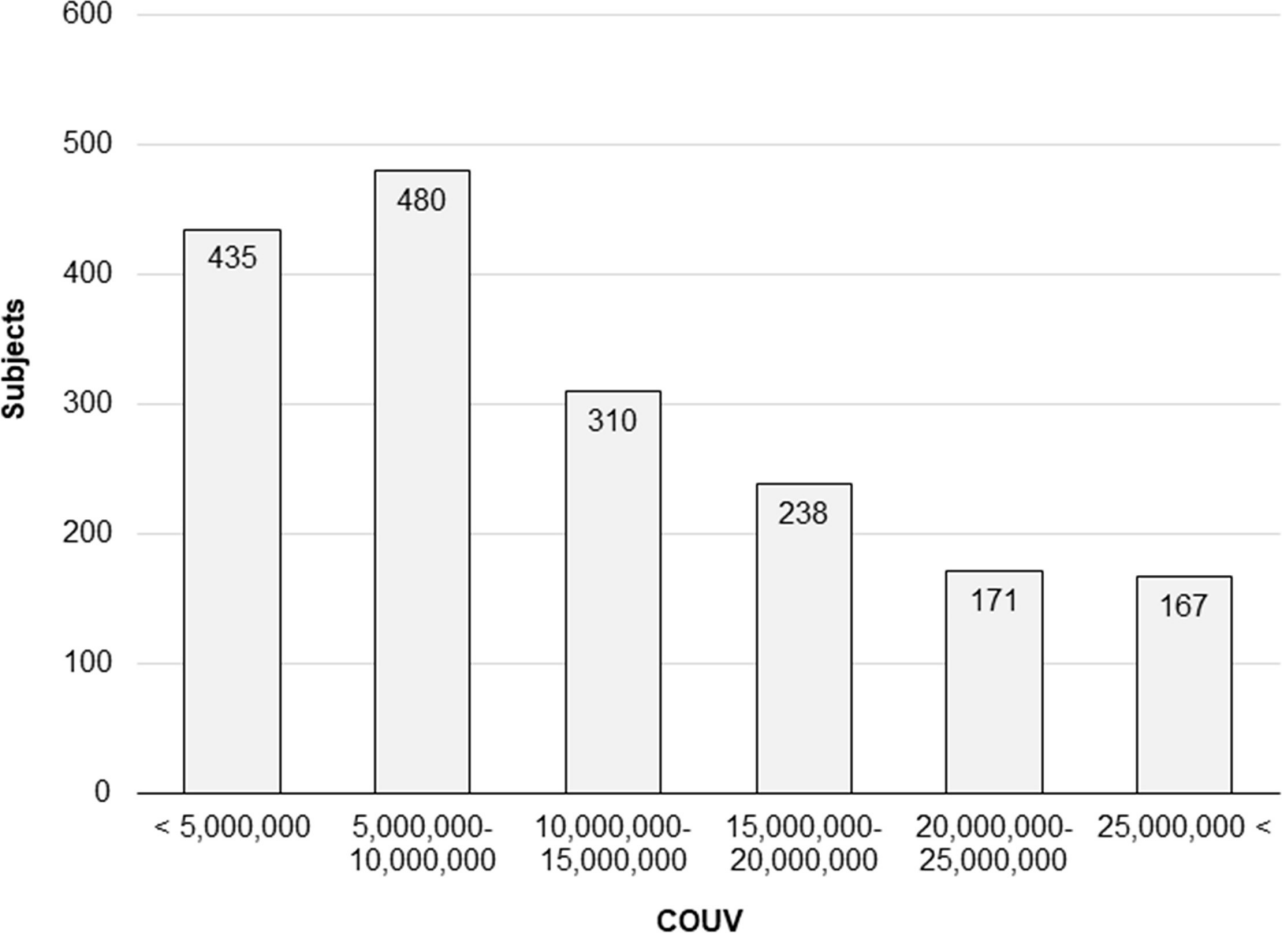
COUV distribution among subjects.

Fig 3 shows the COUV distribution for all subjects, and Table 3 shows the mean COUV values stratified by age and sex for all three regions. Approximately one-fourth of the subjects had a COUV of 5 million or lower. This group comprised subjects who spent relatively little time outdoors or who always used glasses/sunglasses and a hat while outdoors. Furthermore, approximately one-fourth of the subjects had a COUV level ranging from 5 to 10 million. Thus, half of the subjects had a COUV level of 10 million or lower. Most subjects in Sanya and Taiyuan were involved in agricultural work, whereas only approximately 6% of the subjects in Taichung were agricultural workers. Females in Taiyuan and Taichung tended to have lower COUV levels than males. However, there was no significant difference in COUV levels by gender in Sanya; in fact, COUV was slightly higher for females in this region. COUV level was the lowest among both sexes aged under 60 years old in Taichung, where UV intensity is stronger than that in Taiyuan. Because Taichung is more urban than rural Taiyuan, individuals possibly spent less time outdoors and were more likely to use glasses in Taichung. However, among individuals aged above 60 years old, COUV levels were approximately the same in Taichung and Taiyuan.

**Table 3:** Mean cumulative ocular UV exposure by region (stratified by sex and age).

	Sanya UV intensity: 233 J/m^2^		Taiyuan UV intensity: 142 J/m^2^		Taichung UV intensity: 213 J/m^2^	
	Ave	SD	Ave	SD	Ave	SD
Sex						
M	20,485,574	6,907,481	10,260,275	5,414,556	8,967,806	7,823,783
F	21,963,246	6,470,755	8,962,699	4,843,394	6,434,134	4,765,471
Age (y)						
≤59	18,282,224	4,171,967	9,050,005	4,093,863	6,214,076	4,503,783
60–69	22,826,454	5,776,211	9,628,754	5,477,093	9,294,882	7,757,357
70–79	26,954,518	7,107,816	9,962,456	6,526,622	12,491,506	9,873,043
≥80	30,769,901	7,140,611	12,360,888	7,137,888	11,098,765	7,027,718

We divided the subjects into two groups of approximately 900 individuals each based on whether their COUV level was more (high COUV group) or less (low COUV group) than 10 million, which was the median COUV level for all subjects. Next, we investigated the risk of developing the five cataract types in the two groups. We also investigated the risk of developing each of the five cataract types using the mean COUV level in Japanese people (approximately 14,000,000, among residents aged 50 years old and above in Monzen, Ishikawa prefecture, 36°48’ N, 136°46’ E, 127 J/m^2^) as a reference; COUV2 represented twice this value, whereas COUV3 and COUV4 were three- and four-fold greater, respectively.

### Risk of developing the five types of cataract with increased COUV level

Compared with the low COUV group, the odds ratio of cataract of the high COUV group was 5.35 (95% CI: 3.73–7.67) for NUC, 1.87 (1.09–3.19) for PSC, and 1.35 (1.01–1.80) for RD, indicating a significantly increased risk of developing the three types (Table 4). However, COR exhibited no significant associations with CEN− or CEN+. Similarly, we reported no significant association for WCs.

**Table 4:** Risk of the five types of cataract in the high cumulative ocular UV exposure (COUV) group compared with the low COUV group (adjusted for age, sex, axial length, and DM).

Risk factor		Odds ratio	(95% CI)	p value	
Age (y)^§^		1.01	(0.99–1.02)	0.165	
Sex	M	1.00			
	F	0.58	(0.46–0.72)	<0.001	**
COR	Without COR	1.00			
	With CEN-	1.09	(0.77–1.54)	0.642	
	With CEN+	0.74	(0.48–1.15)	0.180	
NUC	Without NUC	1.00			
	With NUC	5.35	(3.37–7.67)	<0.001	**
PSC	Without PSC	1.00			
	With PSC	1.87	(1.09–3.19)	0.022	*
RD	Without RD	1.00			
	With RD	1.35	(1.01–1.80)	0.045	*
WC	Without WC	1.00			
	With WC	0.78	(0.52–1.18)	0.237	

^§^ Odds for age indicates the risk for each additional one year of age.

* p < 0.05

** p < 0.01.

An investigation of the odds ratio as the mean COUV that increased from two- to four-fold (COUV2, COUV3, COUV4) for the reference revealed that for COUV2, the risk significantly increased for NUC (1.90, 1.11–3.25), whereas it significantly decreased for WC (0.53, 0.34–0.84) (Table 5). For COUV3, the risk significantly increased for NUC (7.98, 4.66–13.68), whereas it significantly decreased for COR (CEN+) (0.49, 0.25–0.93) and WC (0.50, 0.28–0.89). Lastly, for COUV4, significant increases were observed for NUC (11.89, 5.49–25.76) and RD (2.09, 1.02–4.28). The risk of developing NUC increased with greater UV exposure, as shown by the odds ratios of 1.9-, 7.98-, and 11.89-fold for COUV2, COUV3, and COUV4, respectively. However, COR (CEN−) did not exhibit any significant associations with increased COUV level. The risk of developing COR (CEN+) in COUV3 and WC in COUV2 and COUV3 exhibited an inverse relationship with increased COUV level; this risk was calculated to be approximately 0.5-fold.

**Table 5:** Risk of the five types of cataract for two-, three- and four-fold cumulative ocular UV exposure (COUV) based on the mean COUV in Japanese people (all adjusted for age, sex, axial length, and DM).

Risk factor	Odds ratio	(95% CI)		p-value	
COUV2 (2-fold)	Age (y) ^§^	1.02	(1.01–1.04)	0.005	**
	Sex (F) ^¶^	0.61	(0.48–0.78)	<0.001	**
	COR (CEN-)	0.87	(0.57–1.33)	0.515	
	COR (CEN+)	0.94	(0.58–1.52)	0.797	
	NUC	1.90	(1.11–3.25)	0.019	*
	PSC	0.74	(0.37–1.51)	0.412	
	RD	0.95	(0.67–1.34)	0.762	
	WC	0.53	(0.34–0.84)	0.007	**
COUV3 (3-fold)	Age (y) ^§^	1.01	(0.99–1.03)	0.162	
	Sex (F) ^¶^	0.68	(0.50–0.92)	0.013	*
	COR (CEN-)	1.06	(0.65–1.72)	0.826	
	COR (CEN+)	0.49	(0.25–0.93)	0.028	*
	NUC	7.98	(4.66–13.68)	<0.001	**
	PSC	1.84	(0.91–3.71)	0.088	
	RD	1.25	(0.83–1.88)	0.280	
	WC	0.50	(0.28–0.89)	<0.001	**
COUV4 (4-fold)	Age (y) ^§^	1.16	(1.12–1.21)	<0.001	**
	Sex (F) ^¶^	0.58	(0.32–1.08)	0.085	
	COR (CEN-)	1.16	(0.55–2.48)	0.695	
	COR (CEN+)	0.68	(0.27–1.71)	0.414	
	NUC	11.89	(5.49–25.76)	<0.001	**
	PSC	1.05	(0.40–2.79)	0.924	
	RD	2.09	(1.02–4.28)	0.044	*
	WC	0.74	(0.33–1.64)	0.458	

^§^Odds ratio for age indicates the risk for each additional 1 year of age.

^¶^Odds ratio for sex indicates the F risk when M is 1.00.

* p < 0.05

** p < 0.01.

### Risk of developing COR (CEN−, CEN+) accompanying COUV increase considering WC comorbidity

Table 6 shows the risk of developing COR in the high COUV group compared with the low COUV group, considering WC comorbidity. Cases with opacity within the central 3-mm diameter area of the pupil were designated as CEN+, and cases with ring-shaped opacity showed no opacity in the center of the pupils; therefore, all of these cases were designated as CEN−. In eyes with WC, the risk of ring-shaped opacity was significantly elevated (1.86, 1.04–3.32) and that of wedge-shaped opacity (CEN+) significantly decreased (0.56, 0.37–0.86). In cases of COR with no WC, the risks of axle-shaped opacity (CEN−) (2.07, 1.03 − 4.18) and ring-shaped opacity (2.96, 1.46–5.99) were significantly increased. The risk of wedge-shaped opacity (CEN+) was significantly decreased (0.59, 0.36–0.95), even with no WC. The risk of ring-shaped opacity significantly increased with greater COUV regardless of WC. Meanwhile, CEN+, in which opacity was present in the center of the pupil, exhibited no association or a decreased risk with increased COUV.

**Table 6:** COR (axle-shaped, wedge-shaped, and ring-shaped) risk of the high cumulative ocular UV exposure (COUV) group compared with the low COUV group (all adjusted for age, sex, axial length, and DM).

Risk factor		Odds ratio	(95% CI)	p value	
WC present	COR axle-shaped CEN-	1.46	(0.82–2.60)	0.196	
	COR axle-shaped CEN+	0.62	(0.37–1.04)	0.071	
	COR wedge-shaped CEN-	0.90	(0.62–1.31)	0.585	
	COR wedge-shaped CEN+	0.56	(0.37–0.86)	0.008	**
	COR ring-shaped CEN-	1.86	(1.04–3.32)	0.036	*
	COR ring-shaped CEN+	-	-	-	
No WC	COR axle-shaped CEN-	2.07	(1.03–4.18)	0.041	*
	COR axle-shaped CEN+	0.59	(0.33–1.08)	0.085	
	COR wedge-shaped CEN-	0.97	(0.64–1.47)	0.886	
	COR wedge-shaped CEN+	0.59	(0.36–0.95)	0.030	*
	COR ring-shaped CEN-	2.96	(1.46–5.99)	0.003	**
	COR ring-shaped CEN+	-	-	-	

* p < 0.05

** p < 0.01

We investigated associations between ocular UV exposure and risk of cataract in Han people living in regions of China and Taiwan with varying UV intensities. Our investigation of the risk associated with increased COUV levels indicated that after adjusting for age, sex, and other factors related to cataract onset such as diabetes [11], and AL [30], and NUC was found to be the cataract type with the highest risk associated with increased COUV level. Some epidemiological studies have reported no association between UV exposure and NUC onset [9, 12], in contrast, others have suggested an association between UV exposure and NUC onset [13–17]. Although our data does not differentiate between exposure dose by frequency at the regions studied, our results are supported by those of Giblin et al. [8] who reported that NUC onset occurs with low long-term UVA exposure using a guinea pig model; our results also agree with a report indicating that NUC prevalence is markedly high in tropical and subtropical regions exposed to high UV intensity [13]. Our results showed that greater COUV was associated with an increased odds ratio for NUC, with a risk increase of approximately 12-fold for COUV4, which indicated high exposure. These results suggest that high UV exposure is a risk factor for NUC onset. COUV4, which indicated high exposure, was also associated with an approximately two-fold increased risk of developing RD. Thus, RD may also be associated with UV exposure. RD often accompanies NUC [31, 32], suggesting that these disease types share common risk factors.

Our investigation of COUV and risk of COR in all cases indicated no significant risk increase with greater COUV level. Many reports have indicated that the amount of ocular UV exposure is associated with COR [6–11]. However, these reports differ from our investigation because they were all conducted in mid- and high-latitude regions that receive relatively moderate UV intensity. Our study investigated people living in Taiyuan, located in a middle latitude region; Taichung, located in a subtropical region; and Sanya, located in a tropical region. In addition, because no reports have yet investigated the relationship between UV intensity and development of cataract among Han people, the risk of developing COR due to UV might differ between Caucasians, who are inherently at a high risk of developing NUC, and Han people, who are at a high risk of developing COR. In Caucasians, who are at a high risk of developing NUC, UV possibly has a limited effect on NUC but might markedly affect COR. Conversely, in Han people, who are at high risk of developing COR, UV possibly has a limited effect on COR but might markedly affect NUC. However, when divided by type into axle-, wedge-, and ring-shaped opacity, the investigation of COR indicated a significant positive association with COUV in ring-shaped opacity cases regardless of WC. A significant association was also observed for cases without WC among eyes exhibiting axle-shaped opacity around the pupil region. UV exposure-related COR opacity is often localized around the pupil region, with opacity particularly developing from the lower nasal side [33]. Upon entering the eye, UV induces the “Coroneo effect” whereby light is refracted by the cornea and is concentrated on the nasal side [34]. Thus, UV exposure-related conditions such as pterygium often develop on the nasal side [35]. No significant association was observed between UV exposure dose and axle- and wedge-shaped opacities that progress to the center of the pupil region. Significant decreased risk regardless of presence of WC in eyes with wedge-shaped opacity in the center of the pupil region was associated with UV exposure dose. Significant risk decreases were also observed for WC in the group receiving high-dose UV. In many cases of COR in which the opacity was localized in the center of the pupil, there was a high possibility of opacity caused by WC. Thus, if UV exposure is not a risk factor for WC onset, it is highly possible that UV is also not a risk factor for COR development from the central pupil from WC. The underlying mechanism of WC onset is unknown. However, a lens change arises from Y-shaped suture breaks. We believe that the possibility cannot be denied that lens shape changes due to changes such as adjustments of the lens when the eye shifts depth of focus place physical stress on the Y-shaped suture lines, which could lead to WC development. For individuals who primarily engage in close-proximity work, such as reading, sewing, and desk work, the risk of developing WC may be higher. Moreover, the fact that these people also engage in less outdoor work may explain the negative association observed between WC and COUV. Because no previous studies have investigated COR type, ours is the first to demonstrate that UV exposure risk might differ by COR type. Even if it progresses, ring-shaped opacity does not reach the center of the pupil. Regarding overall COR, COR arising around the lens appears to be significantly associated with UV exposure.

Our investigation of PSC indicated that significant risk increases were observed in the high UV exposure groups than in the low UV exposure groups. However, after dividing the subjects into four groups by COUV level, there were no significant associations. The small sample size of PSC cases may have affected the precision of the analysis. Many reports have indicated that there is no association between PSC onset and UV exposure [10, 11]. The question of whether high levels of UV exposure such as those investigated in this study affect PSC onset requires further investigation with a larger sample size.

This study has some limitations. First, although we investigated cataract risk after adjusting for risk based on AL and diabetes, diabetic history data was based on self-reports and not blood tests. Therefore, this result possibly has low reliability. Second, according to other risk factors for cataract onset such as smoking [12, 30, 36], steroid use [12, 37, 38], ionizing radiation exposure [39–42], and academic background [12, 36, 42], although we included questions about smoking and use of steroids responses to these items were not provided by many subjects, perhaps because they didn’t smoke or use steroids, but we cannot be sure. Therefore, these factors were removed from our adjustment considering their low reliability. We did not question the subjects regarding radiation exposure. Moreover, because many of the subjects had a rural background, we removed the question on academic background from our parameters. Lastly, to assess UV exposure, we questioned the subjects regarding their time spent on outdoor activities in the past, and after adjusting for glasses (sunglasses) and hat use, we calculated COUV levels in terms of individual mean time spent on activities outdoors. However, because these results were based on the subjects’ memories, they could have low reliability. Moreover, because outdoor activities are likely to differ greatly throughout a person’s life as they progress from childhood to young adulthood, middle age, and old age, the subjects must be questioned for each age range. There are, however, significant limitations to the precision of recall bias. A recent report suggested that pinguecula developing in the limbus cornea as a result of UV exposure could be an index for the UV exposure dose a person has received [43]. Because pterygium is also an index for high UV exposure, it could also be effective to conduct future investigations using the presence/absence and extent of pinguecula and pterygium as indexes for the ocular UV exposure level.

In conclusion, the results suggest that increased COUV level among Han people living in areas with varying UV intensities could be a risk factor for developing NUC, RD, and COR (ring-shaped opacity or opacity around the lens equator).

## Supporting information

S1 Table Demographics of the cross-sectional samples examined for the risk of the five types of cataract in the high cumulative ocular UV exposure (COUV) group compared with the low COUV group [Table 4]. (DOCX)

S2 Table Demographics of the cross-sectional samples examined for the risk of the five types of cataract for two-, three- and four-fold cumulative ocular UV exposure (COUV) based on the mean COUV in Japanese people [Table 5]. (DOCX)

S3 Table Demographics of the cross-sectional samples examined for the COR (axle-shaped, wedge-shaped, and ring-shaped) risk of the high cumulative ocular UV exposure (COUV) group compared with the low COUV group [Table 6]. (DOCX)

## References

[1] Hollows F, Moran D. Cataract—the ultraviolet risk factor. Lancet. 1981;2(8258):1249–50. 10.1016/S0140-6736(81)91490-2 . 6118668

[2] Wittenberg S. Solar radiation and the eye: a review of knowledge relevant to eye care. Am J Optom Physiol Opt. 1986;63(8):676–89. 10.1097/00006324-198608000-00012 . 3532811

[3] Taylor HR. Ultraviolet radiation and the eye: an epidemiologic study. Trans Am Ophthalmol Soc. 1989;87:802–53. . 2562534 PMC1298564

[4] Hightower K, McCready J. Comparative effect of UVA and UVB on cultured rabbit lens. Photochem Photobiol. 1993;58(6):827–30. 10.1111/j.1751-1097.1993.tb04978.x . 8310004

[5] Michael R, Soderberg PG, Chen E. Long-term development of lens opacities after exposure to ultraviolet radiation at 300 nm. Ophthalmic Res. 1996;28(4):209–18. 10.1159/000267905 . 8878183

[6] Dillon J, Zheng L, Merriam JC, Gaillard ER. The optical properties of the anterior segment of the eye: implications for cortical cataract. Exp Eye Res. 1999;68(6):785–95. 10.1006/exer.1999.0687 . 10375442

[7] Mody VC Jr, Kakar M, Elfving A, Soderberg PG, Lofgren S. Ultraviolet radiation-B-induced cataract in albino rats: maximum tolerable dose and ascorbate consumption. Acta Ophthalmol Scand. 2006;84(3):390–5. 10.1111/j.1600-0420.2006.00640.x . 16704705

[8] Giblin FJ, Leverenz VR, Padgaonkar VA, Unakar NJ, Dang L, Lin LR, et al UVA light in vivo reaches the nucleus of the guinea pig lens and produces deleterious, oxidative effects. Exp Eye Res. 2002;75(4):445–58. 10.1006/exer.2002.2039 . 12387792 PMC6472706

[9] Taylor HR, West SK, Rosenthal FS, Munoz B, Newland HS, Abbey H, et al Effect of ultraviolet radiation on cataract formation. N Engl J Med. 1988;319(22):1429–33. 10.1056/NEJM198812013192201 . 3185661

[10] West SK, Duncan DD, Munoz B, Rubin GS, Fried LP, Bandeen-Roche K, et al Sunlight exposure and risk of lens opacities in a population-based study: the Salisbury Eye Evaluation project. JAMA. 1998;280(8):714–8. 10.1001/jama.280.8.714 . 9728643

[11] McCarty CA, Nanjan MB, Taylor HR. Attributable risk estimates for cataract to prioritize medical and public health action. Invest Ophthalmol Vis Sci. 2000;41(12):3720–5. . 11053268

[12] Delcourt C, Carriere I, Ponton-Sanchez A, Lacroux A, Covacho MJ, Papoz L. Light exposure and the risk of cortical, nuclear, and posterior subcapsular cataracts: the Pathologies Oculaires Liees a l'Age (POLA) study. Arch Ophthalmol. 2000;118(3):385–92. 10.1001/archopht.118.3.385 . 10721962

[13] Sasaki H, Jonasson F, Shui YB, Kojima M, Ono M, Katoh N, et al High prevalence of nuclear cataract in the population of tropical and subtropical areas. Dev Ophthalmol. 2002;35:60–9. 10.1159/000060806 . 12061279

[14] Hayashi LC, Hayashi S, Yamaoka K, Tamiya N, Chikuda M, Yano E. Ultraviolet B exposure and type of lens opacity in ophthalmic patients in Japan. Sci Total Environ. 2003;302(1–3):53–62. S0048-9697(02)00320-0. . 12526897 10.1016/s0048-9697(02)00320-0

[15] Zigman S, Datiles M, Torczynski E. Sunlight and human cataracts. Invest Ophthalmol Vis Sci. 1979;18(5):462–7. . 437948

[16] Neale RE, Purdie JL, Hirst LW, Green AC. Sun exposure as a risk factor for nuclear cataract. Epidemiology. 2003;14(6):707–12. 10.1097/01.ede.0000086881.84657.98 . 14569187

[17] Pastor-Valero M, Fletcher AE, de Stavola BL, Chaques-Alepuz V. Years of sunlight exposure and cataract: a case-control study in a Mediterranean population. BMC Ophthalmol. 2007;7:18 10.1186/1471-2415-7-18 . 18039367 PMC2234085

[18] Bochow TW, West SK, Azar A, Munoz B, Sommer A, Taylor HR. Ultraviolet light exposure and risk of posterior subcapsular cataracts. Arch Ophthalmol. 1989;107(3):369–72. 10.1001/archopht.1989.01070010379027 . 2923558

[19] Deane JS, Hall AB, Thompson JR, Rosenthal AR. Prevalence of lenticular abnormalities in a population-based study: Oxford Clinical Cataract Grading in the Melton Eye Study. Ophthalmic Epidemiol. 1997;4(4):195–206. 10.3109/09286589709059193 . 9500154

[20] Frost NA, Sparrow JM, Moore L. Associations of human crystalline lens retrodots and waterclefts with visual impairment: an observational study. Invest Ophthalmol Vis Sci. 2002;43(7):2105–9. . 12091403

[21] Yan Q, Sasaki H, Qu J, Yamashita H, Kojima M, Honda R, et al Population Based Survey of Ocular Diseases in the Farmers with Low Income; a Preliminary Study in the Rural Area of Liaoning Province, China [Japanese]. J Kanazawa Med Univ. 2005;30(4):535–42.

[22] Qu J, Sasaki H, Liu Z, Zhou J, Wu K, Kojima M, et al Current status about blindness among low-income farmers in Sanya District, Hainan, China [Japanese]. Rinsho Ganka (Jpn J Clin Ophthalmol). 2007;61(3):411–6.

[23] Sasaki K, Yamashiro Y, Kojima M, Honda T, Sakamoto Y, Cheng HM, et al Assessment of pinguecula and pterygium in the aging eye with ultraviolet fluorescence photography (UVFP): Data analysis of eye disease survey in Shanxi, China. Rehabilitation science: memoirs of the Tohoku Bunka Gakuen University Faculty of Medical Science & Welfare, Department of Rehabilitation. 2009;5(1):31–40.

[24] Thylefors B, Chylack LT Jr., Konyama K, Sasaki K, Sperduto R, Taylor HR, et al A simplified cataract grading system. Ophthalmic Epidemiol. 2002;9(2):83–95. . 11821974 10.1076/opep.9.2.83.1523

[25] Gaballa S, Allam R, Abouhussein N, Raafat K. IOL master and A-scan biometry in axial length and intraocular lens power measurements. Delta J Ophthalmol. 2017;18(1):13–9. 10.4103/1110-9173.201623

[26] Wong L, Ho SC, Coggon D, Cruddas AM, Hwang CH, Ho CP, et al Sunlight exposure, antioxidant status, and cataract in Hong Kong fishermen. J Epidemiol Community Health. 1993;47(1):46–9. 10.1136/jech.47.1.46 . 8436894 PMC1059710

[27] Rosenthal FS, Phoon C, Bakalian AE, Taylor HR. The ocular dose of ultraviolet radiation to outdoor workers. Invest Ophthalmol Vis Sci. 1988;29(4):649–56. . 3356520

[28] Ono M. Studies on ultraviolet radiation and health effects: ocular exposure to ultraviolet radiation. Dev Ophthalmol. 2002;35:32–9. 10.1159/000060808 . 12061277

[29] McPeters R, Bhartia PK, Krueger A, Herman J, Wellemeyer C, Seftor C, et al Total Ozone Mapping Spectrometer (TOMS) Level-3 Data Products User's Guide. NASA/TP-2000-209896. 2000:1–31.

[30] Wu Z, Lim JI, Sadda SR. Axial length: a risk factor for cataractogenesis. Ann Acad Med Singapore. 2006;35(6):416–9. . 16865193

[31] Shun-Shin GA, Bron AJ, Brown NP, Sparrow JM. The relationship between central nuclear scatter and perinuclear retrodots in the human crystalline lens. Eye. 1992;6 (Pt 4):407–10. 10.1038/eye.1992.84 . 1478315

[32] Tan AG, Tay WT, Mitchell P, Sandar M, Aung T, Saw SM, et al Prevalence of lens opacities in Asian Malays. Ophthalmic Epidemiol. 2012;19(6):380–7. 10.3109/09286586.2012.733479 . 23171207

[33] Sasaki H, Kawakami Y, Ono M, Jonasson F, Shui YB, Cheng HM, et al Localization of cortical cataract in subjects of diverse races and latitude. Invest Ophthalmol Vis Sci. 2003;44(10):4210–4. . 14507863 10.1167/iovs.01-1221

[34] Maloof AJ, Ho A, Coroneo MT. Influence of corneal shape on limbal light focusing. Invest Ophthalmol Vis Sci. 1994;35(5):2592–8. . 8163347

[35] McKnight CM, Sherwin JC, Yazar S, Forward H, Tan AX, Hewitt AW, et al Pterygium and conjunctival ultraviolet autofluorescence in young Australian adults: the Raine study. Clin Experiment Ophthalmol. 2014 10.1111/ceo.12455 . 25307729

[36] Yang M, Zhu R, Liang C, Liu B, Qi Y, Zhang J, et al [Cataract risk factor survey in Funing county of Jiangsu province]. Zhonghua Yan Ke Za Zhi. 2014;50(3):179–83. 10.3760/cma.j.issn.0412-4081.2014.03.005 . 24841812

[37] Praveen MR, Shah GD, Vasavada AR, Mehta PG, Gilbert C, Bhagat G. A study to explore the risk factors for the early onset of cataract in India. Eye (Lond). 2010;24(4):686–94. 10.1038/eye.2009.137 . 19521430

[38] Wang JJ, Rochtchina E, Tan AG, Cumming RG, Leeder SR, Mitchell P. Use of inhaled and oral corticosteroids and the long-term risk of cataract. Ophthalmology. 2009;116(4):652–7. 10.1016/j.ophtha.2008.12.001 . 19243828

[39] Jacob S, Boveda S, Bar O, Brezin A, Maccia C, Laurier D, et al Interventional cardiologists and risk of radiation-induced cataract: results of a French multicenter observational study. Int J Cardiol. 2013;167(5):1843–7. 10.1016/j.ijcard.2012.04.124 . 22608271

[40] Mrena S, Kivela T, Kurttio P, Auvinen A. Lens opacities among physicians occupationally exposed to ionizing radiation—a pilot study in Finland. Scand J Work Environ Health. 2011;37(3):237–43. 10.5271/sjweh.3152 . 21340441

[41] Vano E, Kleiman NJ, Duran A, Rehani MM, Echeverri D, Cabrera M. Radiation cataract risk in interventional cardiology personnel. Radiat Res. 2010;174(4):490–5. 10.1667/RR2207.1 . 20726724

[42] Tang Y, Wang X, Wang J, Jin L, Huang W, Luo Y, et al Risk factors of age-related cataract in a Chinese adult population: the Taizhou Eye Study. Clin Exp Ophthalmol. 2018;46(4):371–9. 10.1111/ceo.13040 . 28842942

[43] Ip MH, Chui JJ, Tat L, Coroneo MT. Significance of Fuchs Flecks in Patients With Pterygium/Pinguecula: Earliest Indicator of Ultraviolet Light Damage. Cornea. 2015;34(12):1560–3. 10.1097/ICO.0000000000000621 . 26398157

