# Correction: Association between ultraviolet radiation exposure dose and cataract in Han people living in China and Taiwan: A cross-sectional study

**DOI:** 10.1371/journal.pone.0218857

**Published:** 2019-06-20

**Authors:** Hisanori Miyashita, Natsuko Hatsusaka, Eri Shibuya, Norihiro Mita, Mai Yamazaki, Teppei Shibata, Hidetoshi Ishida, Yuki Ukai, Eri Kubo, Hiroshi Sasaki

There is an error in Table 5. The column headings “Odds ratio” and “(95% CI)” are incorrectly placed. The third column should be “Odds ratio” and the fourth column should be “(95% CI)”.

**Table 5:** Risk of the five types of cataract for two-, three- and four-fold cumulative ocular UV exposure (COUV) based on the mean COUV in Japanese people (all adjusted for age, sex, axial length, and DM).

Risk factor		Odds ratio	(95% CI)	p-value
COUV2 (2-fold)	Age (y) ^§^	1.02	(1.01–1.04)	0.005**
	Sex (F) ^¶^	0.61	(0.48–0.78)	<0.001**
	COR (CEN-)	0.87	(0.57–1.33)	0.515
	COR (CEN+)	0.94	(0.58–1.52)	0.797
	NUC	1.90	(1.11–3.25)	0.019*
	PSC	0.74	(0.37–1.51)	0.412
	RD	0.95	(0.67–1.34)	0.762
	WC	0.53	(0.34–0.84)	0.007**
COUV3 (3-fold)	Age (y) ^§^	1.01	(0.99–1.03)	0.162
	Sex (F) ^¶^	0.68	(0.50–0.92)	0.013*
	COR (CEN-)	1.06	(0.65–1.72)	0.826
	COR (CEN+)	0.49	(0.25–0.93)	0.028*
	NUC	7.98	(4.66–13.68)	<0.001**
	PSC	1.84	(0.91–3.71)	0.088
	RD	1.25	(0.83–1.88)	0.280
	WC	0.50	(0.28–0.89)	<0.001**
COUV4 (4-fold)	Age (y) ^§^	1.16	(1.12–1.21)	<0.001**
	Sex (F) ^¶^	0.58	(0.32–1.08)	0.085
	COR (CEN-)	1.16	(0.55–2.48)	0.695
	COR (CEN+)	0.68	(0.27–1.71)	0.414
	NUC	11.89	(5.49–25.76)	<0.001**
	PSC	1.05	(0.40–2.79)	0.924
	RD	2.09	(1.02–4.28)	0.044*
	WC	0.74	(0.33–1.64)	0.458

^§^ Odds ratio for age indicates the risk for each additional 1 year of age.

^¶^ Odds ratio for sex indicates the F risk when M is 1.00.

* p < 0.05,

** p < 0.01
