# Correction: Association between ultraviolet radiation exposure dose and cataract in Han people living in China and Taiwan: A cross-sectional study

**DOI:** 10.1371/journal.pone.0222679

**Published:** 2019-09-12

**Authors:** The PLOS ONE Staff

There are errors in Tables 2 and 4. The first column of Table 2 should be labeled “Sanya”, not “Taiyuan.” Additionally, the values in the first and second columns of Table 2 are incorrect. In Table 4, there is an error in the value listed in the second column of the NUC With NUC row. Please see the correct Tables 2 and 4 here. The publisher apologizes for the errors.

**Table 2:** Prevalence of the five types of cataract per region.

	Sanya UV intensity: 233 J/m^2^	Taiyuan UV intensity: 142 J/m^2^	Taichung UV intensity: 213 J/m^2^
COR (CEN-)	17.6% (14.0–21.1)	12.9% (10.3–15.5)	8.3% (6.2–10.3)
COR (CEN+)	7.6% (5.1–10.8)	10.1% (7.7–12.4)	6.7% (4.9–8.5)
NUC	43.6% (39.0–48.1)	7.2% (5.2–9.2)	6.6% (4.8–8.4)
PSC	12.9% (9.8–16.0)	3.0% (1.6–4.3)	3.4% (2.0–4.7)
RD	40.7% (36.1–45.2)	22.3% (19.1–25.6)	16.2% (13.5–18.9)
WC	4.7% (2.7–6.6)	7.2% (5.2–9.2)	11.9% (9.5–14.3)

() = 95% confidence interval for overall prevalence

**Table 4:** Risk of the five types of cataract in the high cumulative ocular UV exposure (COUV) group compared with the low COUV group (adjusted for age, sex, axial length, and DM).

Risk factor		Odds ratio	(95% CI)	p-value	
Age (y) ^§^		1.01	(0.99–1.02)	0.165	
Sex	M	1.00			
	F	0.58	(0.46–0.72)	<0.001	**
COR	Without COR	1.00			
	With CEN-	1.09	(0.77–1.54)	0.642	
	With CEN+	0.74	(0.48–1.15)	0.180	
NUC	Without NUC	1.00			
	With NUC	5.35	(3.73–7.67)	<0.001	**
PSC	Without PSC	1.00			
	With PSC	1.87	(1.09–3.19)	0.022	*
RD	Without RD	1.00			
	With RD	1.35	(1.01–1.80)	0.045	*
WC	Without WC	1.00			
	With WC	0.78	(0.52–1.18)	0.237	

^§^ Odds for age indicates the risk for each additional one year of age.

* p < 0.05,

** p < 0.01
