# Supplementary material for: Association between ultraviolet radiation exposure dose and cataract in Han people living in China and Taiwan: A cross-sectional study
Source: PLoS One. 2019 Apr 25;14(4):e0215338. doi: 10.1371/journal.pone.0215338 (PMC6483175; doi:10.1371/journal.pone.0215338)
Supplement: S1 Table — (DOCX) [file pone.0215338.s001.docx]

**S1 Table 1.** **Demographics of the cross-sectional samples examined for the risk of the five types of cataract in the high cumulative ocular UV exposure (COUV) group compared with the low COUV group [Table 4]**

|  | | COUV group | | | |
| --- | --- | --- | --- | --- | --- |
|  |  | Low  [COUV ave: 6,250,438 ± 2,985,866] | | High  [COUV ave: 20,818,676 ± 6,023,476] | |
|  |  | N | % | N | % |
| N |  | 1132 | 62.9 | 669 | 37.1 |
| Age | (ave ± SD; y) | (56.4 ± 9.6) | | (61.3 ± 10.2) | |
| Sex | M | 408 | 36.0 | 292 | 25.8 |
|  | F | 724 | 64.0 | 377 | 33.3 |
| AL | (ave ± SD; mm) | (23.56 ± 1.47) | | (23.17 ± 1.04) | |
| DM | No | 1032 | 91.2 | 639 | 95.5 |
|  | Yes | 100 | 8.8 | 30 | 4.5 |
| COR | Without COR | 936 | 82.7 | 499 | 74.6 |
|  | With CEN- | 108 | 9.5 | 112 | 16.7 |
|  | With CEN+ | 88 | 7.8 | 58 | 8.7 |
| NUC | Without NUC | 1060 | 93.6 | 452 | 67.6 |
|  | With NUC | 72 | 6.4 | 217 | 32.4 |
| PSC | Without PSC | 1098 | 97.0 | 602 | 90.0 |
|  | With PSC | 34 | 3.0 | 67 | 10.0 |
| RD | Without RD | 942 | 83.2 | 418 | 62.5 |
|  | With RD | 190 | 16.8 | 251 | 37.5 |
| WC | Without WC | 1039 | 91.8 | 610 | 91.2 |
|  | With WC | 93 | 8.2 | 59 | 8.8 |

COUV = cumulative ocular UV exposure, N = number, ave = average, SD = standard deviation,

M = male, F = female, AL = axial length, DM = diabetes mellitus, COR = cortical cataract,

CEN- = opacity absence in the central 3-mm diameter area of the pupil,

CEN+ = opacity presence in the central 3-mm diameter area of the pupil,

NUC = nuclear cataract, PSC = posterior subcapsular cataract, RD = retrodots, WC = waterclefts
