# Supplementary material for: Association between ultraviolet radiation exposure dose and cataract in Han people living in China and Taiwan: A cross-sectional study
Source: PLoS One. 2019 Apr 25;14(4):e0215338. doi: 10.1371/journal.pone.0215338 (PMC6483175; doi:10.1371/journal.pone.0215338)
Supplement: S2 Table — (DOCX) [file pone.0215338.s002.docx]

**S2 Table 2. Demographics of the cross-sectional samples examined for the risk of the five types of cataract for two-, three- and four-fold cumulative ocular UV exposure (COUV) based on the mean COUV in Japanese people [Table 5]**

|  | | COUV group | | | | | | | |
| --- | --- | --- | --- | --- | --- | --- | --- | --- | --- |
|  |  | Group 1  (reference) | | Group 2  (2-fold) | | Group 3  (3-fold) | | Group 4  (4-fold) | |
|  |  | N | % | N | % | N | % | N | % |
| N |  | 576 | 32.0 | 556 | 30.9 | 428 | 23.8 | 241 | 13.4 |
| Age | (ave ± SD; y) | (55.0 ± 10.0) | | (57.9 ± 9.0) | | (58.0 ± 8.9) | | (67.1 ± 9.8) | |
| Sex | M | 212 | 36.8 | 196 | 35.3 | 197 | 46.0 | 95 | 39.4 |
|  | F | 364 | 63.2 | 360 | 64.7 | 231 | 54.0 | 146 | 60.6 |
| AL | (ave ± SD; mm) | (24.05 ± 1.60) | | (23.04 ± 1.12) | | (23.16 ± 1.09) | | (23.20 ± 0.95) | |
| DM | No | 524 | 91.0 | 508 | 91.4 | 407 | 95.1 | 232 | 96.3 |
|  | Yes | 52 | 9.0 | 48 | 8.6 | 21 | 4.9 | 9 | 3.7 |
| COR | Without COR | 481 | 83.5 | 455 | 81.8 | 343 | 80.1 | 156 | 64.7 |
|  | With CEN- | 52 | 9.0 | 56 | 10.1 | 53 | 12.4 | 59 | 24.5 |
|  | With CEN+ | 43 | 7.5 | 45 | 8.1 | 32 | 7.5 | 26 | 10.8 |
| NUC | Without NUC | 545 | 94.6 | 515 | 92.6 | 357 | 83.4 | 95 | 39.4 |
|  | With NUC | 31 | 5.4 | 41 | 7.4 | 71 | 16.6 | 146 | 60.6 |
| PSC | Without PSC | 554 | 96.2 | 544 | 97.8 | 403 | 94.2 | 199 | 82.6 |
|  | With PSC | 22 | 3.8 | 12 | 2.2 | 25 | 5.8 | 42 | 17.4 |
| RD | Without RD | 489 | 84.9 | 453 | 81.5 | 315 | 73.6 | 103 | 42.7 |
|  | With RD | 87 | 15.1 | 103 | 18.5 | 113 | 26.4 | 138 | 57.3 |
| WC | Without WC | 521 | 90.5 | 518 | 93.2 | 398 | 93.0 | 212 | 88.0 |
|  | With WC | 55 | 9.5 | 38 | 6.8 | 30 | 7.0 | 29 | 12.0 |

COUV = cumulative ocular UV exposure, N = number, ave = average, SD = standard deviation,

M = male, F = female, AL = axial length, DM = diabetes mellitus, COR = cortical cataract,

CEN- = opacity absence in the central 3-mm diameter area of the pupil,

CEN+ = opacity presence in the central 3-mm diameter area of the pupil,

NUC = nuclear cataract, PSC = posterior subcapsular cataract, RD = retrodots, WC = waterclefts
