# Supplementary material for: Association between ultraviolet radiation exposure dose and cataract in Han people living in China and Taiwan: A cross-sectional study
Source: PLoS One. 2019 Apr 25;14(4):e0215338. doi: 10.1371/journal.pone.0215338 (PMC6483175; doi:10.1371/journal.pone.0215338)
Supplement: S3 Table — (DOCX) [file pone.0215338.s003.docx]

**S3 Table 3. Demographics of the cross-sectional samples examined for the COR (axle-shaped, wedge-shaped, and ring-shaped) risk of the high cumulative ocular UV exposure (COUV) group compared with the low COUV group [Table 6]**

|  | | | COUV group | | | |
| --- | --- | --- | --- | --- | --- | --- |
|  |  |  | Low  [COUV ave: 6,250,438  ± 2,985,866] | | High  [COUV ave: 20,818,676  ± 6,023,476] | |
|  |  |  | N | % | N | % |
| N |  |  | 1132 | 62.9 | 669 | 37.1 |
| Age |  | (ave ± SD; y) | (56.4 ± 9.6) | | (61.3 ± 10.2) | |
| Sex |  | M | 408 | 36.0 | 292 | 25.8 |
|  |  | F | 724 | 64.0 | 377 | 33.3 |
| AL |  | (ave ± SD; mm) | (23.56 ± 1.47) | | (23.17 ± 1.04) | |
| DM |  | No | 1032 | 91.2 | 639 | 95.5 |
|  |  | Yes | 100 | 8.8 | 30 | 4.5 |
| COR | WC present | Without axle-shaped CEN-/+ | 1060 | 93.6% | 602 | 90.0% |
|  |  | With axle-shaped CEN- | 24 | 2.1% | 38 | 5.7% |
|  |  | With axle-shaped CEN+ | 48 | 4.2% | 29 | 4.3% |
|  |  | Without wedge-shaped CEN-/+ | 959 | 84.7% | 545 | 81.5% |
|  |  | With wedge-shaped CEN- | 93 | 8.2% | 77 | 11.5% |
|  |  | With wedge-shaped CEN+ | 80 | 7.1% | 47 | 7.0% |
|  |  | Without ring-shaped CEN-/+ | 1113 | 98.3% | 628 | 93.9% |
|  |  | With ring-shaped CEN- | 19 | 1.7% | 41 | 6.1% |
|  |  | With ring-shaped CEN+ | 0 | 0.0% | 0 | 0.0% |

|  | | | COUV group | | | |
| --- | --- | --- | --- | --- | --- | --- |
|  |  |  | Low  [COUV ave: 6,300,458  ± 2,997,171] | | High  [COUV ave: 20,593,223  ± 5,828,065] | |
|  |  |  | N | % | N | % |
| N |  |  | 1038 | 63.0% | 610 | 37.0% |
| Age |  | (ave ± SD; y) | (55.6 ± 9.3) | | (60.3 ± 9.9) | |
| Sex |  | M | 369 | 35.5% | 264 | 43.3% |
|  |  | F | 669 | 64.5% | 346 | 56.7% |
| AL |  | (ave ± SD; mm) | (23.57 ± 1.50) | | (23.18 ± 1.06) | |
| DM |  | No | 956 | 92.1% | 586 | 96.1% |
|  |  | Yes | 82 | 7.9% | 24 | 3.9% |
| COR | No WC | Without axle-shaped CEN-/+ | 993 | 95.7% | 557 | 91.3% |
|  |  | With axle-shaped CEN- | 13 | 1.3% | 31 | 5.1% |
|  |  | With axle-shaped CEN+ | 32 | 3.1% | 22 | 3.6% |
|  |  | Without wedge-shaped CEN-/+ | 917 | 88.3% | 510 | 83.6% |
|  |  | With wedge-shaped CEN- | 68 | 6.6% | 63 | 10.3% |
|  |  | With wedge-shaped CEN+ | 53 | 5.1% | 37 | 6.1% |
|  |  | Without ring-shaped CEN-/+ | 1027 | 98.9% | 573 | 93.9% |
|  |  | With ring-shaped CEN- | 11 | 1.1% | 37 | 6.1% |
|  |  | With ring-shaped CEN+ | 0 | 0.0% | 0 | 0.0% |

COUV = cumulative ocular UV exposure, N = number, ave = average, SD = standard deviation,

M = male, F = female, AL = axial length, DM = diabetes mellitus, COR = cortical cataract, WC = waterclefts,

CEN- = opacity absence in the central 3-mm diameter area of the pupil,

CEN+ = opacity presence in the central 3-mm diameter area of the pupil
